# A protocol for a systematic review of birth preparedness and complication readiness programs

**DOI:** 10.1186/2046-4053-2-11

**Published:** 2013-02-08

**Authors:** Andrea Solnes Miltenburg, Yadira Roggeveen, Marianne van Elteren, Laura Shields, Joske Bunders, Jos van Roosmalen, Jelle Stekelenburg

**Affiliations:** 1 Athena Institute for Research on Innovation and Communication in Health and Life sciences, Faculty of Earth and Life Sciences, VU University, De Boelelaan 1105, 1081 HV, Amsterdam, the Netherlands; 2 Department of Medical Humanities (EMGO) Institute for Health and Care Research VU, University Medical Center (VUmc), Van der Boechorststraat 7, 1081, BT Amsterdam, the Netherlands; 3 Department of Obstetrics & Gynaecology, Leeuwarden Medical Centre, Henri Dunantweg 2, 8934, AD Leeuwarden, The Netherlands

**Keywords:** Birth preparedness and complication readiness, Birth plan, Maternal mortality, Utilization, Skilled birth attendant, Safe motherhood, Health education

## Abstract

**Background:**

One of the effective strategies for reducing the number of maternal deaths is delivery by a skilled birth attendant. Low utilization of skilled birth attendants has been attributed to delay in seeking care, delay in reaching a health facility and delay in receiving adequate care. Health workers could play a role in helping women prepare for birth and anticipate complications, in order to reduce delays. There is little evidence to support these birth preparedness and complication readiness (BP/CR) programs; however, BP/CR programs are frequently implemented. The objective of this review is to assess the effect of BP/CR programs on increasing skilled birth attendance in low-resource settings.

**Methods:**

Due to the complexity of BP/CR programs and the need to understand why certain programs are more effective than others, we will combine both quantitative and qualitative studies in this systematic review. Search terms were selected with the assistance of a health information specialist. Three reviewers will independently select and assess studies for quality. Data will be extracted by one reviewer and checked for accuracy and completeness by a second reviewer. Discussion between the reviewers will resolve disagreements. If disagreements remain, a third party will be consulted. Data analysis will be carried out in accordance with the BP/CR matrix, developed by the Johns Hopkins Program for International Education in Gynecology and Obstetrics (JHPIEGO). Study data will be grouped and analyzed by quality and study design and regrouped according to type of intervention strategy.

**Discussion:**

This review will provide: 1) an insight into existing BP/CR programs, 2) recommendations on effective elements of the different approaches, 3) proposals for concrete action plans for health professionals in the field of reproductive health in resource-poor settings and 4) an overview of existing knowledge gaps requiring further research.

**Trial registration:**

PROSPERO registration no.: CRD42012003124

## Background

Poor maternal health, leading to maternal death and severe acute maternal morbidity, remains a major problem, especially in sub-Saharan Africa, where the maternal mortality ratio (MMR) is declining steadily [1]. While a number of countries have made substantial progress in reducing child mortality, the high neonatal mortality rate and its link to obstetric causes is still of great concern [2]. The main direct causes for maternal death and severe acute maternal morbidity are hemorrhage, eclampsia, sepsis, obstructed labor and complications arising from an unsafe abortion [3]. It is assumed that most cases of maternal death and severe acute maternal morbidity can be prevented when births are assisted by skilled birth attendants. Safe Motherhood programs were successful in reducing maternal mortality by placing skilled birth attendants within functioning health systems, which include the availability of or referral to emergency obstetric care services [4]. Packages of (integrated) interventions, including antenatal and postnatal care services, safe abortion services, and the availability of family planning services can further reduce severe acute maternal morbidity and improve overall maternal health. It is expected that there will be a reduction in both neonatal mortality and morbidity rates when these services are available [4-6]. However, the availability of maternal health services does not mean they that are affordable and accessible, provide good quality of care and are used.

The low utilization of maternal health services is frequently analyzed with the Three Delays Model developed by Thaddeus and Maine (1994), which identifies three phases of delay: delay in seeking care, delay in reaching care and delay in receiving adequate care when reaching a health facility [5]. Better knowledge of danger signs means that the predictable elements of the three phases of delay can be anticipated and prepared for with a birth plan for each pregnancy. Birth preparedness and complication readiness (BP/CR) is a process of planning for birth and anticipating actions needed in case of an emergency [7]. In 2001 the Johns Hopkins Program for International Education in Gynecology and Obstetrics (JHPIEGO) developed the BP/CR matrix, which ‘delineates the roles of policymakers, facility managers, providers, communities, families, and women in ensuring that women and newborns receive appropriate, effective, and timely care’ [7]. It is hypothesized that implementation of BP/CR concepts that focus on individuals, families and communities could reduce at least the first two phases of delay. An operational BP/CR matrix means prepared health facilities that are able to handle childbirths and complications, thus contributing to a reduction of the third phase of delay [7,8]. Although there is little evidence that BP/CR interventions are effective, some promising results from a Nepalese study have been published. Components of the birth-preparedness matrix were implemented and led to a 30% reduction in neonatal mortality and 75% reduction in maternal mortality [9]. However, another Nepalese study on the implementation of a birth-preparedness package did not show any change in the utilization of skilled birth attendants. This study concluded that programs that merely encourage pregnant women to use skilled birth attendants were not efficient and suggested research must go beyond the household level in order to have a significant impact [10].

Although there is a paucity of evidence measuring the effect of BP/CR, it has nevertheless been implemented as an essential part of antenatal care consultations. BP/CR is included in the new World Health Organization (WHO) model for antenatal care as part of antenatal care education. Several countries have adopted this new model to fit the local context [11-13]. A growing number of pregnant women make use of antenatal care services. Roughly 80% of the women in sub-Saharan Africa use antenatal care services at least once [14]. The WHO model proposes that antenatal care attendance should result in all pregnant women being aware of the need for skilled birth attendance as well as increased knowledge of how and when to access skilled birth attendants [12]. Despite the growing number of antenatal care visits, the number of births attended by skilled birth attendants still lags behind. In Tanzania, for example, despite the antenatal care coverage rate of around 94% (one time visit), the rate of skilled birth attendance can be as low as 30%, especially in rural areas. The same study found that two components of BP/CR, health education and counseling, were the least likely components of antenatal care to be provided [15].

Evidence for the effect of antenatal care education and BP/CR programs on the reduction of the three phases of delay, ideally resulting in a reduction of maternal mortality and morbidity, is limited [8,12] According to Stanton (2004), reasons for the limited evidence include the use of study samples that are too small to capture the complexity of birth preparedness. Also, the historical focus on collecting data on BP/CR using women as the primary target group has hampered the gaining of insights into the success or failure of interventions [8]. In many rural contexts, women are not the decision-makers in the family and are thus rarely involved in pregnancy-related decisions [14]. To gain insights into the involvement of decision-makers, interventions should include partners and other community members. For example, a woman is only fully prepared when, in addition to having planned where to deliver (preferably with skilled birth attendants), funds are allocated for transport and family members are identified to accompany her when labor starts or complications arise [7,14,16]. How to link individuals, families and communities to health systems that are capable of supporting birth preparedness, requires further study. Examples of links include adequate transportation services and health-care workers capable of responding according to guidelines if there is an obstetric emergency while simultaneously attending to the woman’s needs [10,14,16]. Representative samples of involved actors are needed to evaluate BP/CR interventions. The BP/CR matrix provides an overview of the different roles and responsibilities for the variety of actors implementing BP/CR. So far, the main effect measurements have mostly focused on health outcome indicators, such as mortality and morbidity rates; however, the evaluation of the knowledge, intentions and behaviors of the various actors around childbirth might provide insights into why BP/CR programs are effective or not. Qualitative evaluation of BP/CR programs can assist this process [8].

## Study design

### Aim and objectives

The objective of this study is to assess the effect of BP/CR programs on increasing skilled birth attendance in a low-resource environment. We have chosen to focus on the effects of skilled birth attendance since it is expected that this will give us an indication of the effects of BP/CR before any impact on mortality and morbidity is noticeable, especially since health outcome indicators such as MMR are difficult to obtain with sufficient accuracy to measure progress [17].

As there are several ways to implement and evaluate BP/CR interventions, the following key research questions need to be answered.

1. To what extent do BP/CR programs result in increasing skilled birth attendance.

2. What strategies are used to implement BP/CR?

3. What methodologies are used to measure the effectiveness of BP/CR?

4. Which factors influence the effectiveness of BP/CR?

## Methods

This systematic review follows the guidelines for a systematic review as given in the *Cochrane Handbook for Systematic Reviews of Interventions*[18], the PRISMA statement [19] and the guidelines published by the NHS Center for Reviews and Dissemination [20]. As randomized trials may be scarce in this area, excluding other quantitative data (for example, quasi-experimental studies) and qualitative data would substantially narrow the evidence base and exclude valuable data. Furthermore, quantitative evidence is needed to assess the effectiveness of BP/CR, whereas qualitative data is needed to inform important factors influencing BP/CR effectiveness [21]. Recent literature shows that, although challenging, there are ways to include qualitative studies in systematic reviews [20,22].

### Study inclusion criteria

The studies included are randomized controlled trials, quasi-experimental studies, cohort studies, case control studies, cross-sectional surveys and qualitative studies. Table 1 displays the PICOTS elements: participants, intervention, control, outcome, timeframe and setting.

**Table 1:** Inclusion criteria (PICOTS elements)

**PICOTS**	**Inclusion criteria**
Participants	Pregnant women, women who have recently delivered, husbands of pregnant women, husbands of women who have recently delivered, health-care providers, traditional birth attendants, all adults in the community (in low- and middle-income countries)
Intervention	Antenatal care education containing BP/CR components, community programs including BP/CR, single BP/CR interventions, training of health workers (skilled birth attendant, community health worker, health promotion officer), training of community volunteers
Control	Standard practice
Outcome	Preparedness: Knowledge of danger signs, creation of and applying a birth plan, funds allocated, transportation arrangements
	Pregnancy: Antenatal care with skilled health worker
	Delivery: Delivery by a skilled birth attendant, maternal and neonatal mortality and morbidity
Timeframe	Duration of follow-up and possible exposure to the intervention
Setting	Low- and middle-income countries. Interventions can use facility-based, community-based or home-based services

BP/CR, birth preparedness and complication readiness; PICOTS, participant, intervention, control, outcome, timeframe and setting.

### Participants

We have included women of reproductive age who are pregnant at any given gestational stage or women who have recently given birth. We have restricted inclusion to women who have had births in the past two years to avoid recall problems, since we assume that recollections of pregnancy and birth experience more than two years ago will be prone to bias. Husbands of pregnant women or husbands of women who recently gave birth are also included in the target population. The targeted population also includes health workers who deliver pregnancy care. This includes skilled birth attendants, health promotion officers and community health workers, and others working in community, government or private (including faith-based) health institutions. We also include (trained) traditional birth attendants, because of their important role in childbirth in many communities.

### Intervention and control

Interventions include single interventions that address one component of the BP/CR matrix, such as training of health workers to deliver BP/CR education. Also included are combined interventions such as overall antenatal care interventions and community health interventions that include multiple BP/CR elements. Public health interventions usually consist of a package of components and can be seen as complex since the different components can have independent and inter-dependent effects [20]. In the analysis and presentation of our results, we will mention if BP/CR was part of a sole intervention or part of a combined approach. We expect that many interventions are not defined or described as relating to birth preparedness but in fact do contribute to the process of planning for birth. Since BP/CR comprises elements of antenatal, intrapartum and postpartum care, interventions can take place in all or one of these phases of pregnancy and childbirth. Also interventions can take place at different levels of care (household, community, provider, facility and policy level). Interventions made on one level and those that cover all levels will be included. We anticipate difficulties in defining a control group, since elements of the BP/CR matrix have already been incorporated into standard care. Control groups could receive standard care or interventions that are not BP/CR interventions. Furthermore, control groups are generally highly heterogeneous and depend on the available resources in low- and middle-income countries. In this study, we define standard care as the care that is provided in clinics according to local or national guidelines. However, we acknowledge that due to limited (human) resources these guidelines are not always adhered to [15]. Due to difficulties in performing controlled interventions in rural settings, uncontrolled studies will also be included.

### Outcomes

Studies will be included if they assess any of the primary or secondary outcomes mentioned below. Lower maternal and neonatal mortality might not necessarily be seen as a result of the BP/CR elements alone and are, therefore, chosen as secondary outcomes. Since skilled birth attendance sometimes is presented as a complementary outcome rather than a main outcome measure we also include studies that do not primarily promote the use of skilled birth attendance but contribute to reaching this goal. For example, some facility-based studies focus more on service delivery and quality improvement, which influences health-care utilization indirectly. Although such interventions might not directly result in increased skilled birth attendance, it is assumed that they will contribute to the promotion of the use of skilled birth attendants in the long run. Studies will also be included when the primary outcome is related to the use of (trained) traditional birth attendants.

#### Primary outcome

Delivery by a skilled birth attendant (defined as an accredited health professional such as a midwife, doctor or nurse who has been educated and trained to proficiency in the skills needed to manage uncomplicated pregnancies, childbirth and the immediate postnatal period, and in the identification, management and referral of complications in women and newborns [23]).

#### Secondary outcomes

**Maternal mortality**: The death of a woman while pregnant or within 42 days of termination of pregnancy, irrespective of the duration and site of the pregnancy, from any cause related to or aggravated by the pregnancy or its management, but not from accidental or incidental causes [24].

**Severe acute maternal morbidity or near miss**: A woman who nearly died but survived a complication that occurred during pregnancy, childbirth or within 42 days of termination of pregnancy [25].

**Neonatal mortality**: The death of a neonate divided between early neonatal mortality (death in the first week of life) or late neonatal mortality (death after 7 to 28 days of life) [2].

**Neonatal morbidity or near miss**: A neonate that survived a life-threatening condition at birth or during the neonatal period as a result of adverse influences or treatments (or non-treatments) during the neonatal period [26].

**Knowledge/awareness**: Knowledge of the importance of pregnancy care and delivery care by a skilled birth attendant, the danger signs of pregnancy, the location of health institutions and/or emergency obstetric care and existing community services for emergencies (funds and transport) [8].

**Intention**: The intention to save money for childbirth, to use a skilled birth attendant, to arrange for transport, to contact health facilities when complications arise and to use postpartum care [8].

**Practice/behavior**: Women who had more than one antenatal care visit, a birth plan was made, money was saved, arrangements were made for emergency transport, the birth was attended by a skilled birth attendant [8].

### Timeframe

We will assess the duration of follow-up and possible exposure to the intervention. It is expected that the length of time required for interventions to show any effect from the use of skilled birth attendants could easily exceed 3 to 5 years. We anticipate that improvements in knowledge, intentions and behavior with regard to birth preparedness could be measured earlier, but will ultimately result in improvements in the use of skilled birth attendants [7].

### Setting

We decided to review evidence from populations in low- and middle-income countries as classified by the World Bank [27]. Study settings for interventions can be facility based, community based or home based.

### Search methods

To identify relevant studies, the following three bibliographic databases will be searched: PubMed, Embase and CINAHL (Cumulative Index to Nursing and Allied Health Literature). We will hand search potentially relevant internet sources such as African Index Medicus, African Journals Online and the World Health Organization (WHO) library to increase the likelihood of including studies from low-resource environments. In addition we will check relevant web pages from the WHO, the Population Council and Google Scholar for additional grey literature. All reference lists in retrieved articles will be checked to see if they contain additional relevant studies. The searches will be limited to publications that have been published between 1 January 1987 and 1 October 2012, that are in English and are for low- and middle-income countries. A health information specialist assisted in the selection of search terms. The literature search will use the following keywords in relation to pregnancy: health, knowledge, attitudes, practices; birth preparedness or birth plan; safe motherhood; empowerment; women’s (or maternal) autonomy. Based on a pilot search we excluded ‘complication readiness’, ‘education’ and ‘counseling’ from our search because relevant articles also appeared with the selected keywords and these additional terms were judged to be unnecessary. The preliminary search strategy is given in Additional file 1.

### Study selection

Three reviewers (ASM, YR and ME) will independently search and screen abstracts and titles in duplicate. The titles and abstracts for articles found will be matched against the BP/CR matrix. The full articles will retrieved for all included articles or those that remain unclear and which will be assessed to see if they match the inclusion criteria. Reviewers will independently review the articles to see if they meet the inclusion criteria. Discussion between the reviewers will resolve disagreements. If disagreements remain, a third party (JB, JR or JS) will be consulted. A flow chart showing the number of studies remaining at each stage will be used according to the PRISMA statement [19]. The flow chart is given in Additional file 2.

### Quality criteria

The quality of the included studies will be assessed by the three reviewers independently. Two instruments will be used in the quality assessment of quantitative and qualitative studies. The risk of bias of quantitative studies will be assessed using the criteria outlined in the *Cochrane Handbook for Systematic Reviews of Interventions*[18]. Although there has been considerable debate on how the quality of qualitative research should be assessed, several studies have successfully included qualitative studies along with quantitative studies in systematic reviews [28,29]. Several appraisal tools have been developed. For this research, we will make use of the criteria developed by Walsh and Downe (2006). After reviewing all the existing frameworks and checklists, they developed a workable list of essential criteria classified into eight key areas: scope and purpose, design, sampling strategy, analysis, interpretation, reflexivity, ethical dimensions, and relevance and transferability [30]. All articles based on qualitative data will be assessed according to these eight criteria and will be rated as strong, moderate or weak. See Additional file 3 for a detailed overview of the assessment tool for qualitative studies.

### Data extraction

Study data will be extracted using a standard format and entered into Microsoft Excel spreadsheets. Data will be extracted by one reviewer and checked for accuracy and completeness by a second reviewer. Data to be extracted include identification features of the study (setting, study design, outcomes and funding sources), stakeholder group(s) involved in the intervention (policymakers, facility managers, providers, communities, families and women), whether the intervention is focused on antenatal, intrapartum and/or postpartum care, type of intervention strategy (single or combined interventions) and level of evidence (according to the Oxford levels of evidence [31]).

### Data analysis and synthesis

First, the analysis will use the BP/CR indicators developed by JHPIEGO [32]. The matrix provides an overview of all stakeholders with a shared responsibility for BP/CR such as policymakers, health-care providers and communities. It includes all elements for which individual stakeholders are responsible in either pregnancy, childbirth or the postpartum period. The format of this matrix can be seen in Additional file 4. Study data for each stakeholder will be grouped and analyzed by quality and study design (quantitative or qualitative studies). After this, the study data will be collected and regrouped according to the type of intervention strategy. From this, a descriptive analysis of the included studies will be formulated, identifying those types of intervention that have an effect on primary and secondary outcomes.

We anticipate that there will be substantial heterogeneity between studies regarding both interventions and outcomes. If it is possible to cluster studies and compute an effect size for a number of outcomes for at least three studies, we will conduct a meta-analysis of randomized controlled trials. The meta-analysis will be performed using the Cochrane Review Manager (the Cochrane Collaboration, Copenhagen, Denmark) [33]. If a meta-analysis is conducted, we will consider heterogeneity using the chi-square test for homogeneity with statistical significance *P* < 0.05 and where I^2^ is the percentage of variation between studies due to heterogeneity rather than chance. Inclusion of cluster-randomized controlled trials in the meta-analysis will be analyzed and reported separately from randomized controlled trials. For dichotomous outcomes, we will compute the odds ratio with a confidence interval of 95% to estimate the effect size, and the standardized mean difference for continuous outcome variables.

The aim of this review is to assess the effects of BP/CR programs. However, the effects are not merely outcomes. We are also interested to know why certain programs seem to be more effective than others. Therefore, we also propose to conduct a narrative synthesis, making use of the available qualitative studies. Narrative synthesis can be used in systematic reviews to tell the story behind the numbers and provide a new body of knowledge to explain the effect. To avoid any chance of bias and remain systematic in our approach, we will make use of the narrative syntheses framework described in the guidance report developed by the UK Economic and Social Research Council. A flow chart summarizing the synthesis process is given in Additional file 5[34]. When the qualitative studies support the outcomes of the quantitative studies, we will use triangulation methods. If, however, there is a disconnect we will analyze it and provide advice for future research.

### Dissemination

Skilled birth attendance is an essential element through which maternal and neonatal health problems can be reduced. Several interventions aim to increase the utilization of skilled birth attendance. This review will assess an element of antenatal care that has gained attention in recent years, namely the development of a birth plan. Recent interventions have aimed to raise awareness and the knowledge of mothers, families and communities, stressing that they are responsible for developing a birth plan and demanding skilled birth attendance. The effectiveness of this intervention is promising, although to what extent and why needs to be determined. The knowledge gained from this review should therefore be of interest to those involved in reproductive health matters in low- and middle-income countries, ranging from midwives and clinical officers on the ground, to academic researchers and decision-makers at the policy level. Also communities, families and women will be targeted. We will make use of dissemination strategies such as publishing in relevant peer-reviewed journals and presenting at conferences. To reach midwives and clinical officers on the ground, we will channel the results through those non-governmental organizations interested in our results and through decision-makers. They will be encouraged to forward the message to communities, families and women. Decision-makers will be reached through reproductive health seminars and conferences and through face-to-face discussions of our findings.

## Discussion

### Expected significance of the study

With the growing demand for evidence based interventions of Safe Motherhood programs, this review will add to the evidence base of effective promotion and implementation of BP/CR programs. This review will provide 1) an insight into existing BP/CR programs, 2) recommendations on effective elements within the different approaches, 3) proposals for concrete action plans for health professionals in the field of reproductive health in resource poor settings and 4) an overview of existing knowledge gaps that require further research.

## Abbreviations

BP/CR: birth preparedness and complication readiness;JHPIEGO: John Hopkins Program for International Education in Gynecology and Obstetrics;MMR: Maternal mortality ratio;PICOTS: Participant, intervention, control, outcome, timeframe and setting;WHO: World Health Organization

## Competing interests

The authors declare that they have no competing interests.

## Authors’ contributions

ASM, YR and ME formed the review team and designed the study. LS was consulted on the methodology. JB, JR and JS provided expert advice and assisted with the study design. All authors read and approved the final manuscript.

## Supplementary Material

Additional file 1 **Search strategy on 12 November 2012.** PubMed (12 November 2012). Click here for file

Additional file 2 **PRISMA 2009 flow chart (Moher et al. **[19]**).** Click here for file

Additional file 3 **Quality assessment tool for qualitative studies – based on criteria developed by Walsh and Downe **[30].
Click here for file

Additional file 4 Birth-preparedness and complication-readiness (BP/CR) matrix (adapted from the Maternal and Neonatal Health Program). Click here for file

Additional file 5 **Flow chart for synthesis process (adapted from guidance developed by Popay et al., **[34]**).** Click here for file
