# Supplementary material for: A protocol for a systematic review of birth preparedness and complication readiness programs
Source: Syst Rev. 2013 Feb 8;2:11. doi: 10.1186/2046-4053-2-11 (PMC3599634; doi:10.1186/2046-4053-2-11)
Supplement: Additional file 1 — Search strategy on 12 November 2012. PubMed (12 November 2012). [file 2046-4053-2-11-S1.pdf]

Additional file1

Search strategy 12th of November 2012

PUBMED (12 Nov 2012)

Search	Add to builder	Query	Items found	Time
#8	Add	Search **(#1 AND #2 AND #5)** Filters: **Publication date from 1987/01/01; English**	2645	04:14:39
#7	Add	Search **(#1 AND #2 AND #5)** Filters: **English**	2961	04:12:09
#6	Add	Search **(#1 AND #2 AND #5)**	3267	04:11:55
#5	Add	Search **(#3 OR #4)**	109002	04:11:24
#4	Add	Search **((Birth preparedness[tiab] OR birth plan*[tiab] OR safe motherhood[tiab] OR empower*[tiab] OR women's autonomy[tiab] OR woman's autonomy[tiab] OR women's authority[tiab] OR woman's authority[tiab] OR maternal autonomy[tiab] OR maternal authority[tiab]))**	11831	04:11:14
#3	Add	Search **((("Health Knowledge, Attitudes, Practice"[Mesh] OR "Awareness"[Mesh] OR "Midwifery"[Mesh] OR "Doulas"[Mesh])) OR "Personal Autonomy"[Mesh])**	98402	04:11:03
#2	Add	Search **(("Africa"[Mesh] OR "Caribbean Region"[Mesh] OR "Central America"[Mesh] OR "Latin America"[Mesh] OR "Mexico"[Mesh] OR "South America"[Mesh] OR "Asia"[Mesh] OR "Indian Ocean Islands"[Mesh] OR "Pacific Islands"[Mesh:NoExp] OR "Melanesia"[Mesh] OR "Micronesia"[Mesh] OR "Polynesia"[Mesh]))**	747955	04:10:51
#1	Add	Search **"Pregnancy"[Mesh]**	672597	04:10:39

Embase Session Results (12 Nov 2012)

No.	Query	Results
#8	**#7** AND [english]/lim AND [1987-2013]/py	**2249**
#7	**#1** AND **#2** AND **#6**	**2854**
#6	**#4** OR **#5**	**138774**
#5	**'birth preparedness'**:ab,ti OR (**birth** NEAR/1 **plan***):ab,ti OR **'safe motherhood'**:ab,ti OR **empower***:ab,ti OR (**women*** NEAR/1 **autonomy**):ab,ti OR (**woman*** NEAR/1 **autonomy**):ab,ti OR (**women*** NEAR/1 **authority**):ab,ti OR (**woman*** NEAR/1 **authority**):ab,ti OR **'maternal autonomy'**:ab,ti OR **'maternal authority'**:ab,ti	**13695**
#4	**'attitude to health'**/exp OR **'awareness'**/exp OR **'midwife'**/exp OR **'doula'**/exp OR **'personal autonomy'**/exp	**126352**
#2	**'africa'**/exp OR **'central america'**/exp OR **'south and central america'**/exp OR **'mexico'**/exp OR **'asia'**/exp OR **'indian ocean'**/exp OR **'pacific islands'**/exp	**868289**
#1	**'pregnancy'**/exp	**589184**

Print Search History CINAHL (12 Nov 2012)

#	Query	Results
S7	S1 AND S2 AND S5<br>Limiters - Published Date from: 19870101-; Language: English	658
S6	S1 AND S2 AND S5	700
S5	S3 OR S4	21,313
S4	TI ((“Birth preparedness” OR “birth plan*” OR “safe motherhood” OR “empower*” OR “women's autonomy” OR “woman's autonomy” OR “women's authority” OR “woman's authority” OR “maternal autonomy” OR “maternal authority”)) OR AB ((“Birth preparedness” OR “birth plan*” OR “safe motherhood” OR “empower*” OR “women's autonomy” OR “woman's autonomy” OR “women's authority” OR “woman's authority” OR “maternal autonomy” OR “maternal authority”))	8,794
S3	(MH "Health Knowledge, Attitudes, Practice+") OR (MH "Awareness+") OR (MH "Midwifery+") OR (MH "Doulas+") OR (MH "Personal Autonomy+")	12,693
S2	(MH "Africa+") OR (MH "Caribbean Region+") OR (MH "Central America+") OR (MH "Latin America+") OR (MH "Mexico+") OR (MH "South America+") OR (MH "Asia+") OR (MH "Indian Ocean Islands+") OR (MH "Pacific Islands") OR (MH "Melanesia+") OR (MH "Micronesia+") OR (MH "Polynesia+")	123,449
S1	(MH "Pregnancy+")	85,864

	Search 12 nov. 2012	Ref.Man. nr	
PubMed	2645	1-2645	
Embase	2249	2646-4894	
CINAHL	658	4895-5552	
Totaal	5552		
Na ontdubbeling	3664		
