# Supplementary figures and images for: A protocol for a systematic review of birth preparedness and complication readiness programs
Source: Syst Rev. 2013 Feb 8;2:11. doi: 10.1186/2046-4053-2-11 (PMC3599634; doi:10.1186/2046-4053-2-11)

## ADDITIONAL FILE 2

### PRISMA 2009 Flow Diagram (Moher et al. 2009)

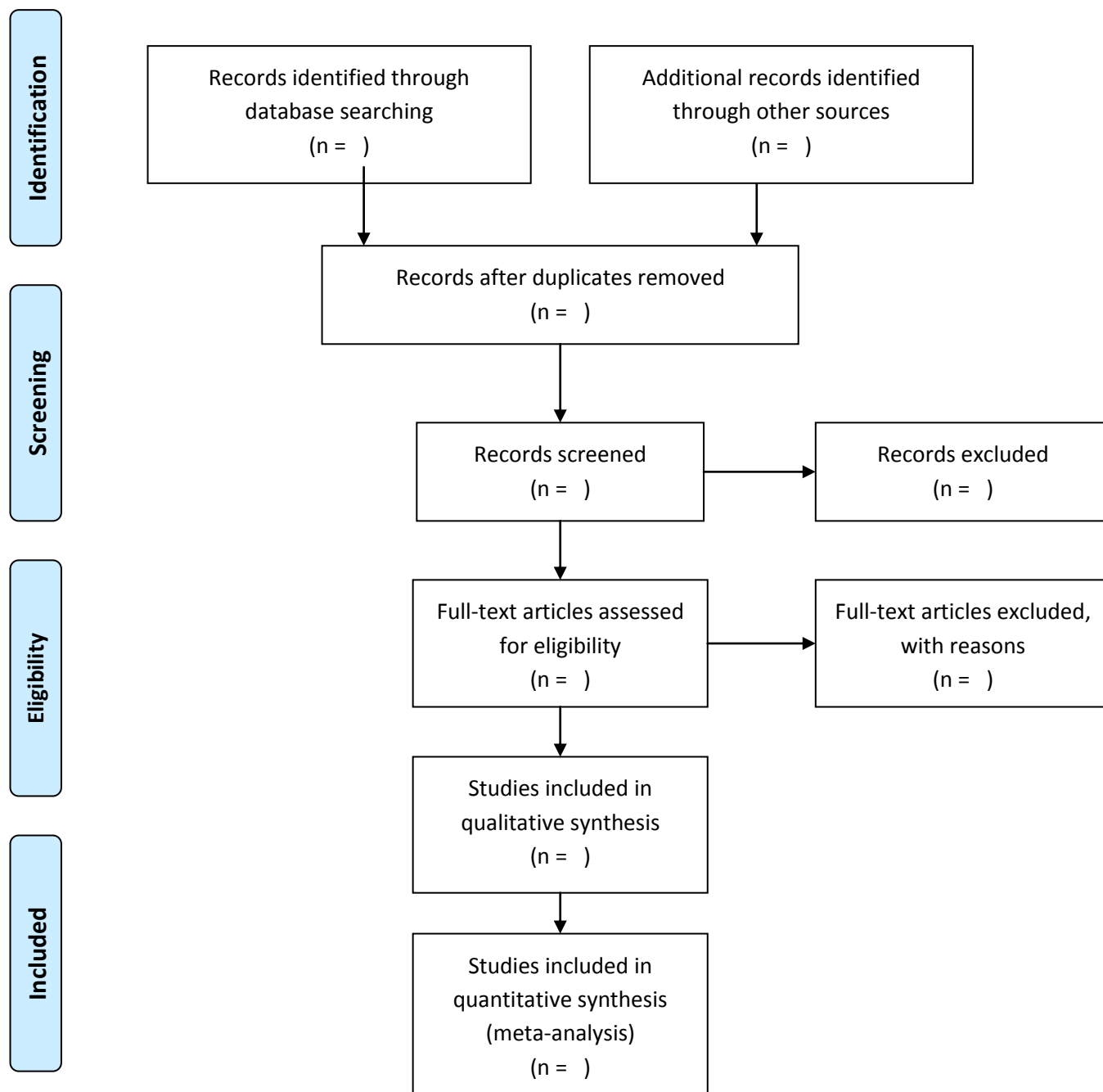

Supplement: Additional file 2 — PRISMA 2009 flow chart (Moher et al. [19]). [file 2046-4053-2-11-S2.pdf]
