# Supplementary material for: A protocol for a systematic review of birth preparedness and complication readiness programs
Source: Syst Rev. 2013 Feb 8;2:11. doi: 10.1186/2046-4053-2-11 (PMC3599634; doi:10.1186/2046-4053-2-11)
Supplement: Additional file 3 — Quality assessment tool for qualitative studies – based on criteria developed by Walsh and Downe [30]. [file 2046-4053-2-11-S3.pdf]

ADDITIONAL FILE 3

QUALITY ASSESSMENT TOOL FOR QUALITATIVE STUDIES – Based on criteria developed by Walsh and Downe (2006)

Ref ID: _________
Author:_________
Year:_________
Reviewer:___________

COMPONENT RATINGS

A) SCOPE AND PURPOSE

(E1) Clear statement of, and rationale for, research question/aims/purposes

No Yes

- Clarity of focus demonstrated
- Explicit purpose given, such as descriptive/explanatory intent, theory building, hypothesis testing
- Link between research and existing knowledge demonstrated

(E2) Study thoroughly contextualized by existing literature

No Yes

- Evidence of systematic approach to literature review, location of literature to contextualize the findings, or both

RATE THIS SECTION	STRONG	MODERATE	WEAK
See dictionary	1	2	3

B) DESIGN

(E1) Method/design apparent, and consistent with research intent

No Yes

Specific prompts

- Rationale given for use of qualitative design
- Discussion of epistemological/ontological grounding
- Rationale explored for specific qualitative method (e.g. ethnography, grounded theory, phenomenology)
- Discussion of why particular method chosen is most appropriate/sensitive/relevant for research question/aims
- Setting appropriate

(E2) Data collection strategy apparent and appropriate

No Yes

Specific prompts

- Were data collection methods appropriate for type of data required and for specific qualitative method?
- Were they likely to capture the complexity/diversity of experience and illuminate context in sufficient detail?

- Was triangulation of data sources used if appropriate?

RATE THIS SECTION	STRONG	MODERATE	WEAK
See dictionary	1	2	3

C) SAMPLING STRATEGY

(E1) Sample and sampling method appropriate
No Yes

Specific prompts

- Selection criteria detailed, and description of how sampling
- was undertaken
- Justification for sampling strategy given
- Thickness of description likely to be achieved from sampling
- Any disparity between planned and actual sample explained

RATE THIS SECTION	STRONG	MODERATE	WEAK
See dictionary	1	2	3

D) ANAYLSIS

(E1) Analytic approach appropriate
No Yes

Specific prompts

- Approach made explicit (e.g. Thematic distillation, constant comparative method, grounded theory)
- Was it appropriate for the qualitative method chosen?
- Was data managed by software package or by hand and why?
- Discussion of how coding systems/conceptual frameworks evolved
- How was context of data retained during analysis
- Evidence that the subjective meanings of participants were portrayed
- Evidence of more than one researcher involved in stages if appropriate to epistemological/theoretical stance
- Did research participants have any involvement in analysis (e.g. member checking)
- Evidence provided that data reached saturation or discussion/rationale if it did not
- Evidence that deviant data was sought, or discussion/rationale if it was not

RATE THIS SECTION	STRONG	MODERATE	WEAK
See dictionary	1	2	3

E) INTEPRETATION

(E1) Context described and taken account of in interpretation
No Yes

Specific prompts

- Description of social/physical and interpersonal contexts of data collection
- Evidence that researcher spent time 'dwelling with the data', interrogating it for competing/alternative explanations of phenomena

(E2) Clear audit trail given

No Yes

Specific prompt

- Sufficient discussion of research processes such that others can follow 'decision trail'

(E3) Data used to support interpretation

No Yes

Specific prompts

- Extensive use of field notes entries/verbatim interview quotes in discussion of findings
- Clear exposition of how interpretation led to conclusions

RATE THIS SECTION	STRONG	MODERATE	WEAK
See dictionary	1	2	3

F) REFLEXIVITY

(E1) Researcher reflexivity demonstrated

No Yes

Specific prompts

- Discussion of relationship between researcher and participants during fieldwork
- Demonstration of researcher's influence on stages of research process
- Evidence of self-awareness/insight
- Documentation of effects of the research on researcher
- Evidence of how problems/complications met were dealt with

RATE THIS SECTION	STRONG	MODERATE	WEAK
See dictionary	1	2	3

G) ETHICAL DIMENSIONS

(E1) Demonstration of sensitivity to ethical concerns

No Yes

Specific prompts

- Ethical committee approval granted
- Clear commitment to integrity, honesty, transparency, equality and mutual respect in relationships with participants
- Evidence of fair dealing with all research participants
- Recording of dilemmas met and how resolved in relation to ethical issues
- Documentation of how autonomy, consent, confidentiality, anonymity were managed

RATE THIS SECTION<br>See dictionary	STRONG<br>1	MODERATE<br>2	WEAK<br>3

H) RELEVANCE AND TRANSFERABILITY

(E1) Relevance and transferability evident

No Yes

Specific prompts

- Sufficient evidence for typicality specificity to be assessed
- Analysis interwoven with existing theories and other relevant explanatory literature drawn from similar settings and studies
- Discussion of how explanatory propositions/emergent theory may fit other contexts
- Limitations/weaknesses of study clearly outlined
- Clearly resonates with other knowledge and experience
- Results/conclusions obviously supported by evidence
- Interpretation plausible and 'makes sense'
- Provides new insights and increases understanding
- Significance for current policy and practice outlined
- Assessment of value/empowerment for participants
- Outlines further directions for investigation
- Comment on whether aims/purposes of research were achieved

RATE THIS SECTION<br>See dictionary	STRONG<br>1	MODERATE<br>2	WEAK<br>3

Global rating

COMPONENT RATINGS

Please transcribe the information from the gray boxed on pages 1-4 onto this page

RATE THIS SECTION	STRONG	MODERATE	WEAK
See dictionary	1	2	3

A SELECTION BIAS

B STUDY DESIGN

RATE THIS SECTION	STRONG	MODERATE	WEAK
See dictionary	1	2	3

C CONFOUNDERS

RATE THIS SECTION	STRONG	MODERATE	WEAK
See dictionary	1	2	3

D BLINDING

RATE THIS SECTION	STRONG	MODERATE	WEAK
See dictionary	1	2	3

E DATA COLLECTION METHODS

RATE THIS SECTION	STRONG	MODERATE	WEAK
See dictionary	1	2	3

F WITHDRAWALS AND DROPOUTS

RATE THIS SECTION	STRONG	MODERATE	WEAK
See dictionary	1	2	3

<u>GLOBAL RATING FOR THIS PATER (circle one)</u>

1	STRONG	(four STRONG ratings with no WEAK ratings)
2	MODERATE	(less than four STRONG ratings and one WEAK rating)
3	WEAK	(two or more WEAK ratings)

With both reviewers discussing the ratings”

Is there a discrepancy between the two reviewers with respect to the component (A-F) ratings?

No Yes

If yes, indicate the reason for discrepancy

1 Oversight
2 Differences in interpretation of criteria
3 Differences in interpretation of study

<u>Final decision of both reviewers (circle one):</u>

1 STRONG
2 MODERATE
3 WEAK
