# Supplementary material for: A protocol for a systematic review of birth preparedness and complication readiness programs
Source: Syst Rev. 2013 Feb 8;2:11. doi: 10.1186/2046-4053-2-11 (PMC3599634; doi:10.1186/2046-4053-2-11)
Supplement: Additional file 4 — Birth-preparedness and complication-readiness (BP/CR) matrix (adapted from the Maternal and Neonatal Health Program). [file 2046-4053-2-11-S4.pdf]

ADDITIONAL FILE 4

Birth Preparedness and Complication Readiness (BP/CR) Matrix (adapted from MNHP)

Policymaker	Facility	Provider	Community	Family	Woman
**Pregnancy**					
Creates an environment that ensures evidence based skilled ANC, adequate recourses, encourages participation in policy making by communities, families, individuals, integrates BP/CR into programs	Is equipped, staffed and managed to provide skilled A with essential drugs and equipment for ANC, follows guidelines has functional emergency system, ensures skilled care 24/7, is gender and culturally sensitive, involves communities and reviews cases	Provides skilled ANC both technical (prevent, screen and treatment of disease) and educational (counseling and education on danger signs, health promotion), assists and promotes formulation of birth plan, respects community expectations and educates about BP/CR	Advocates for skilled ANC, facilitates BP/CR actions through education of community members about birth preparedness, has a functional (financial, practical) system, acts according to plans, and advocates through dialogue with providers and policy makers	Supports pregnant woman's birth plans, advocates for skilled ANC, recognizes danger signs and acts according to plan, has knowledge on available (financial, practical) systems and knows how to access them	Prepares for birth with at least 4 ANC visits, develops a birth plan, has knowledge on available (financial, practical) systems, recognizes danger signs and acts according to the plan
**Labor and childbirth**					
Promotes improved care during labor, ensures evidence based skilled care policies, supports policies for management of complications, ensures adequate levels of resources and protocols, encourages participation	Has essential drugs and equipment, follows guidelines on appropriate management of labor, has appropriate space for delivery, functional emergency system, ensures skilled care 24/7, is gender and culturally sensitive, involves communities and reviews cases	Provides skilled care during labor and childbirth (use of parthograph emotional and physical support, clean and safe delivery, recognizes complications, referral when indicated), supports and respects community expectations and educates about BP/CR	Advocates for skilled provider at birth, makes sure woman has labor support, assists in transportation, recognizes danger signs, supports mother/baby friendly decision making, is in dialogue with and supports facility	Recognizes normal labor, advocates for skilled healthcare, facilitates BP/CR plan, supports decision for transport or referral, has access to (financial, practical) systems, purchases necessary medicines and materials	Recognized normal labor, danger signs, knows BP/CR plan, transportation system and has access to personal savings or existing emergency funds
**Postpatum and newborn**					
Promotes improved care during postpartum and newborn care, ensures evidence based skilled care policies, supports policies for management of complications, ensures adequate levels of resources and protocols, encourages participation	Has essential equipment and drugs, follows guidelines on postpartum and newborn care, functional emergency system, ensures 24.7 care, is gender and culturally sensitive, involves communities and reviews cases	Provides skilled postpartum and newborn care (recognizes complications, promotes health and prevents disease in woman and newborn, provides counseling and education, referral if needed), supports and respects community expectations and educates about BP/CR	Advocates for skilled provider in postpartum period, makes sure woman is not alone in postpartum period, recognizes danger signs, supports transportation, functioning blood donation system, educates about complication readiness, is in dialogue with facility	Supports use of postpartum care, recognizes danger signs, agrees with woman decision making process, knows transportation system, supports provider and woman in referral, has knowledge on available (financial, practical) systems and knows how to access them	Seeks postpartum and newborn care at least twice, recognized danger signs, has knowledge on available (financial, practical) systems, recognizes danger signs and acts according to the plan
