# Supplementary material for: A protocol for a systematic review of birth preparedness and complication readiness programs
Source: Syst Rev. 2013 Feb 8;2:11. doi: 10.1186/2046-4053-2-11 (PMC3599634; doi:10.1186/2046-4053-2-11)

ADDITIONAL FILE 5

FLOW CHART FOR SYNTHESIS PROCESS (adapted from guidance developed by Popay et al, 2006)

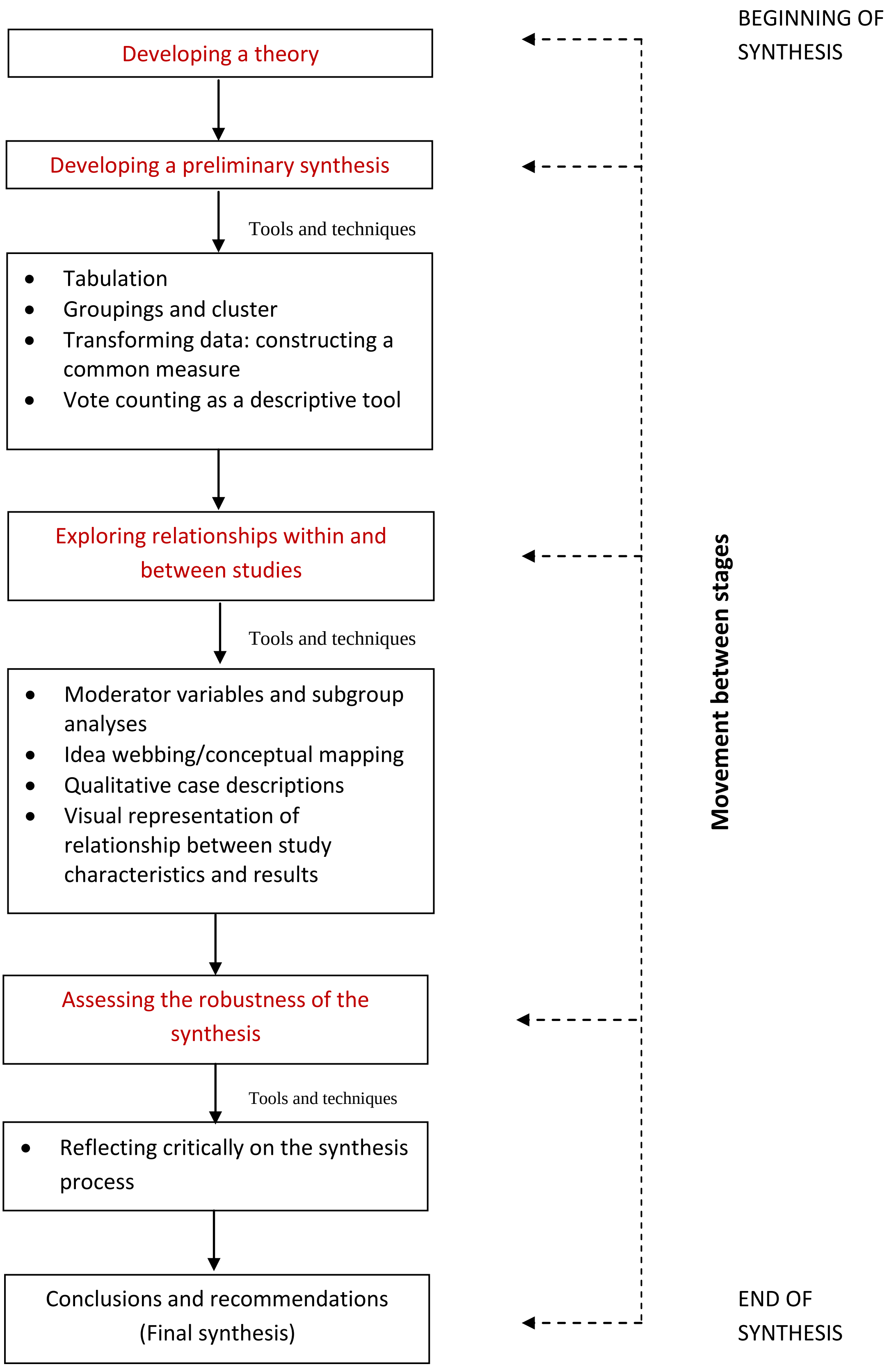

Supplement: Additional file 5 — Flow chart for synthesis process (adapted from guidance developed by Popay et al., [34]). [file 2046-4053-2-11-S5.pdf]
